# Liver Transplantation as a Salvage Therapy Option in Colorectal Liver Metastases: Feasibility, Oncologic Outcomes, and Survival After Failure of Conventional Therapy—A Systematic Review and Meta-Analysis

**DOI:** 10.3390/cancers18081254

**Published:** 2026-04-15

**Authors:** Faiza Hashim Soomro, Tehreem Fatima Kazmi, Mehwish Ansar, Nadia Gulnaz, Rabia Arshad, Gulla Aiste

**Affiliations:** 1 Mid Yorkshire Teaching Trust, Aberford Rd., Wakefield WF1 4DG, UK; nadiagulnaz@gmail.com (N.G.); rabia.arshad79@gmail.com (R.A.); 2 New City Teaching Hospital, New Mirpur City 10250, Pakistan; tehreemqazmi@gmail.com; 3 The Shrewsbury and Telford Hospital NHS Trust, Shrewsbury SY3 8XQ, UK; mehwish0052@gmail.com; 4 Clinic of Surgery, Gastroenterology and Nephrourology, Institute of Clinical Medicine, Faculty of Medicine, Vilnius University, LT-03101 Vilnius, Lithuania; aistegulla@gmail.com

**Keywords:** carcinoembryonic antigen, colorectal neoplasms, disease-free survival, liver neoplasms, liver transplantation, prognosis

## Abstract

**Introduction:** Liver transplantation has re-emerged as a potential therapeutic option for patients with unresectable colorectal liver metastases after failure of standard treatments. This systematic review and meta-analysis evaluated survival outcomes, recurrence patterns, and prognostic factors associated with this approach. **Materials and Methods:** A systematic review was conducted according to PRISMA 2020 guidelines and registered in PROSPERO. Electronic databases were searched for studies published between November 2015 and November 2025, that assessed liver transplantation in the context of unresectable colorectal liver metastases. Random-effect meta-analyses were conducted to estimate the pooled overall survival, disease-free survival and recurrence rates. Heterogeneity was assessed using I^2^ statistics. **Results:** Twenty-three studies involving patients with unresectable liver-only colorectal metastases were included. Pooled overall survival after liver transplantation was 96.6% at 1 year (95% CI 93.9–99.4; I^2^ = 44.3%), 73.4% at 3 years (95% CI 62.9–83.9; I^2^ = 95.4%), and 49.4% at 5 years (95% CI 35.4–63.3; I^2^ = 90.5%). Ten-year overall survival was approximately 27%. The pooled recurrence rate was 63.5% (95% CI 52.5–76.8), and the type of recurrence was mainly extrahepatic, most commonly pulmonary. Disease-free survival was 64.1% (95% CI 47.5–80.7) with substantial heterogeneity (I^2^ = 95.6%). Biological risk factors, including carcinoembryonic antigen levels, metabolic tumor volume, and composite risk scores, consistently influenced survival outcomes. **Conclusions:** In highly selected patients with unresectable colorectal liver metastases, liver transplantation is associated with favorable long-term survival despite frequent recurrence. Outcomes appear to be primarily driven by tumor biology rather than tumor burden, supporting the cautious use within specialized centers under structured selection protocols.

## Simple Summary

Colorectal cancer often spreads to the liver, and many patients are not eligible for curative resection. Recently, liver transplantation has re-emerged as a potential treatment for carefully selected patients with advanced disease where multiple treatment regimens have already been exhausted. In this study, we systematically reviewed and analyzed current evidence to evaluate outcomes following liver transplantation in this setting. We were surprised to see that our carefully chosen patients could have good long-term outcomes, even though their chances of recurrence were high. We believe that outcomes are largely determined by tumor biology and sensitivity to treatment, rather than size or number, which means selecting appropriate patients is crucial. Unfortunately, this area lacks evidence, is extremely selective, and therefore should be considered an evolving and investigational approach. Further prospective research is needed to clarify its role in modern cancer treatment.

## 1. Introduction

Colorectal cancer is the third most commonly diagnosed neoplasm in the world and is still ranked the second-highest cause of cancer-related deaths [1]. Every year, it is estimated that about 1.23 million new cases are reported [2]. More than 50% of patients acquire distant metastases during the disease process, with the liver being the most common site of spread because of direct portal venous drainage of the colon and rectum [3]. Synchronous colorectal liver metastases (CRLMs) are seen in 20–25% of patients at presentation, and a further 30% develop metachronous hepatic metastases [4].

Surgery is the only modality that has curative potential with 5- and 10-year overall survival of around 38% and 26%, respectively [5]. However, despite the high frequency of hepatic involvement, only about 20% of patients with CRLMs are resectable at diagnosis [5,6]. Although advances in systemic therapy and multidisciplinary management have improved outcomes in metastatic colorectal cancer [7], survival remains highly stage-dependent: the 5-year survival rate approaches 91% for localized disease, 36% for regional lymph node involvement, and declines to 14% in the setting of distant metastases [2].

Patients with nonresectable CRLM (NRCRLM) have unfavorable 5-year survival rates of less than 10% [8,9]. Recurrence is very common even after complete liver resection, and this is most likely to happen within the liver (50–75% of recurrences occur in the liver) [10]. Such a high recurrence rate explains why more effective local and systemic therapeutic measures are needed. Since the results following liver transplantation have been improving over the past decades, most notably due to developments in immunosuppression, transplantation has become a possible therapeutic method in patients with unresectable CRLM [11]. Although CRLM historically constituted a contraindication to liver transplantation (LT), early prospective experiences from Norway demonstrated encouraging overall and disease-free survival outcomes in patients with nonresectable CRLM (NRCRLM) undergoing LT [12,13]. A U.S. series likewise reported a 3-year disease-free survival of 53% and overall survival of 60% following LT for CRLM [14]. Most recently, a randomized controlled trial has demonstrated an impressive survival benefit, in which 5-year overall survival was 57% in the transplant arm compared to 13% in patients treated with chemotherapy alone [15].

These new data, along with the advances in living donor liver transplantation (LDLT), machine perfusion technologies, and improved selection criteria, have revived interest in LT as a potentially groundbreaking treatment for unresectable CRLM. There is still an active discussion on the best way to select candidates, the ethical issues surrounding the allocation of organs, and the relative efficacy of transplantation and other liver-focused interventions [16,17].

This systematic review and meta-analysis aims to facilitate the synthesis of modern evidence on the use of LT to treat unresectable CRLM, in particular post-transplant survival rates, recurrence, and prognostic variables that can potentially be applied to patient selection.

## 2. Materials and Methods

### 2.1. Study Design and Eligibility Criteria

This systematic review was conducted in accordance with the Preferred Reporting Items for Systematic Reviews and Meta-Analyses (PRISMA) 2020 guidelines [18] (Tables S1 and S2). The study protocol was prospectively registered in the International Prospective Register of Systematic Reviews (PROSPERO) under the registration number CRD420251174625.

Eligible studies included adult patients aged 18 years or older with a diagnosis of colorectal liver metastases who were considered unresectable or nonresectable and who had failed, progressed on, or were deemed unsuitable for standard treatment modalities, including systemic chemotherapy, hepatic resection, and locoregional therapies. Studies in which liver transplantation was used as the main treatment approach were included, covering deceased donor, living donor, partial liver transplantation, and procedures involving extended-criteria donors.

A broad range of study designs was considered eligible in order to capture the evolving and limited evidence base in this field. These included randomized controlled trials, non-randomized controlled trials, prospective and retrospective cohort studies, case–control studies, prospective or retrospective case series with extractable outcome data, pilot or feasibility studies, registry-based analyses, and original research articles reporting patient-level outcomes. Review articles were included only if they contained extractable original outcome data.

Studies were excluded if they involved pediatric populations, animal or pre-clinical research, editorials, single-patient case reports, expert opinions, or narrative commentaries without original patient data. Studies investigating liver transplantation for mixed oncologic indications were excluded unless outcomes specific to colorectal liver metastases were clearly reported. Abstract-only publications and studies without reported clinical outcomes were also excluded.

### 2.2. Search Strategy, Data Sources and Study Selection

The literature review was conducted in ten electronic databases: PubMed, MEDLINE, Embase, Cochrane Library, Scopus, Web of Science, ScienceDirect, Europe PMC, ClinicalTrials.gov, and Google Scholar. Other sources included EBSCO Dissertations and reference lists of the appropriate articles to cover the available literature extensively.

A combination of Medical Subject Headings (MeSH) and free-text words, including colorectal cancer, colorectal liver metastases, liver transplantation, living donor liver transplantation, unresectable or nonresectable liver metastases, and salvage or last-line therapeutic approaches were used as the search strategy. Only papers published between 22 November 2015 to 22 November 2025 were searched. Articles available in languages other than English were excluded.

**Figure 1:**
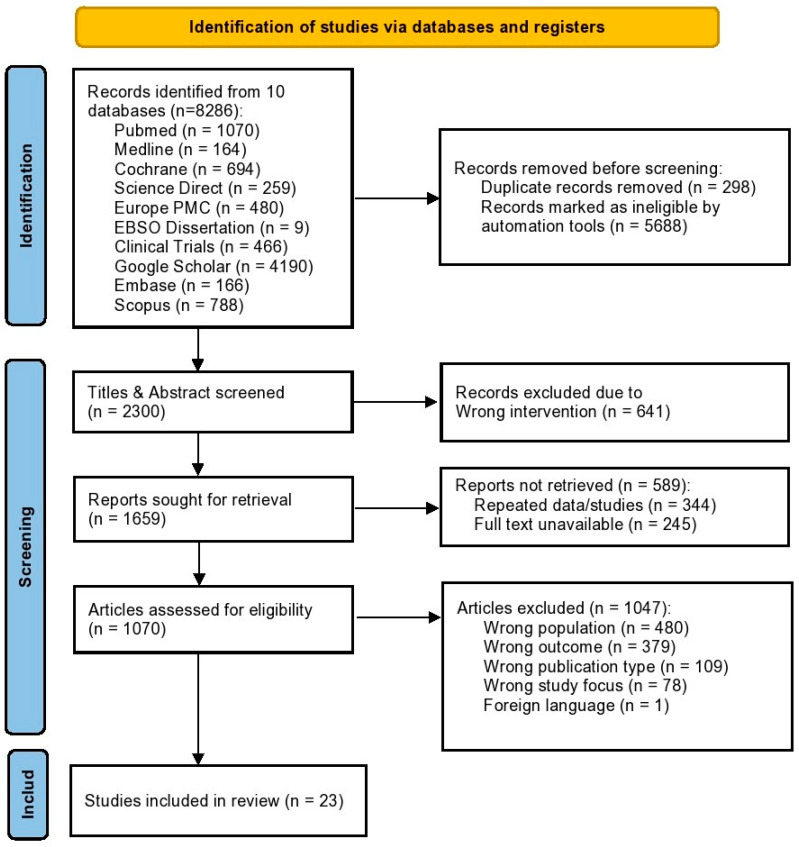
PRISMA flowchart showing the study inclusion process.

The selection of the studies was done in two stages. First, the relevance of titles and abstracts was filtered. Subsequently, full-text articles were located and evaluated in terms of eligibility based on the established inclusion and exclusion criteria. We presented the selection of the studies and the exclusion criteria used at every level in the PRISMA flow diagram (Figure 1).

### 2.3. Data Extraction and Data Tabulation

Two reviewers used a standardized data extraction form (designed in Microsoft Excel) to extract the data, and a third reviewer adjudicated the disagreements with the help of discussion and arbitration. The extracted variables were characteristics of the study identification and design (study design, study period, location, and sample size), characteristics of the patients (age at transplantation and sex distribution), characteristics of the primary colorectal cancer (tumor location and TNM stage), and characteristics of liver metastasis (number of metastases, size of the largest lesion, pre-transplant carcinoembryonic antigen levels, Oslo score, and Fong Clinical Risk Score). Patient records on pre-transplant therapies were also taken, such as prior locoregional therapies, liver resection, and systemic chemotherapy regimens. The outcome-related data included the following: overall survival, disease-free survival, prognostic variables affecting survival, the prognostic value of health-related quality of life at pre-specified post-transplant time intervals, and the prognostic role of pre-transplant baseline symptoms on survival. All the extracted data were tabulated to provide comparisons of data across studies and to support the synthesis of the data using narrative and quantitative methods.

### 2.4. Quality of Evidence Assessment (Risk-of-Bias Assessment)

The Risk-of-Bias in Non-randomized Studies of Interventions (ROBINS-I) tool was used in the methodological quality and risk-of-bias assessment of the included studies. Given that all the included studies consisted of non-randomized prospective and retrospective cohort studies, case series, and observational analyses, ROBINS-I was considered the most appropriate instrument to evaluate potential biases across key methodological domains.

The ROBINS-I assessment had been done on the following domains: bias due to confounding, bias in the selection of participants, bias in the classification of interventions, bias due to deviations from intended interventions, bias due to missing data, bias in measurement of outcomes, and bias in the selection of the reported result. The estimation of the risk-of-bias assessment was visualized with the help of the Risk-of-Bias Visualization (robvis) tool [19].

### 2.5. Non-Randomized Studies (ROBINS-I)

Overall, the non-randomized studies included had moderate to high risks of bias which stems out of the difficulties of assessing the effects of liver transplantation in highly selected patients with unresectable colorectal liver metastases. The largest source of bias among studies was bias caused by confounding, because patient selection to receive liver transplant was heavily based on prognostic variables like tumor burden, chemotherapy response, and biological (e.g., carcinoembryonic antigen levels) and institutional selection criteria. Even though numerous studies tried to adjust confounding by using very stringent eligibility criteria, risk scores (e.g., Oslo score, Fong Clinical Risk Score) or subgroup analysis, they were not able to eliminate residual confounding.

The overall rating of bias in selecting the participants was low to moderate. The adult population was well defined in most studies and engaged unresectable CRLM that had depleted the conventional treatment therapies and were subjected to liver transplantation cases at the specialized centers. In smaller single-center studies and case series, however, it was observed that there could be issues of selective referral patterns, institutional experience, and highly selective transplant candidacy, which restricts generalizability.

Bias in intervention classification was regarded as insignificant in most of the studies because the liver transplantation was well documented and the intervention clear. The type of donor (deceased donor and living donor), graft attributes and methodology of transplant was usually described in adequate detail to reduce misclassification.

The bias on exceptions of planned interventions was also considered to be low since the regime of post-transplant management, such as immunosuppression and surveillance measures, were generally similar in individual studies. The difference between centers and times, however, could have added to differences in outcomes.

The extent of bias (because of missing data) was mostly low-to-moderate. Most studies reported complete follow-up for key outcomes such as overall survival and recurrence. Nonetheless, incomplete reporting of certain prognostic variables, quality-of-life outcomes, and long-term follow-up data was observed in several retrospective analyses.

Bias in outcome measurement was considered low. The overall survival, disease-free survival and recurrence were the objective and clinically relevant outcomes with the least chance of measurement bias. The standardized imaging protocols and histopathological confirmation were usually utilized as radiological evaluations and pathological evaluations of recurrence.

**Figure 2:**
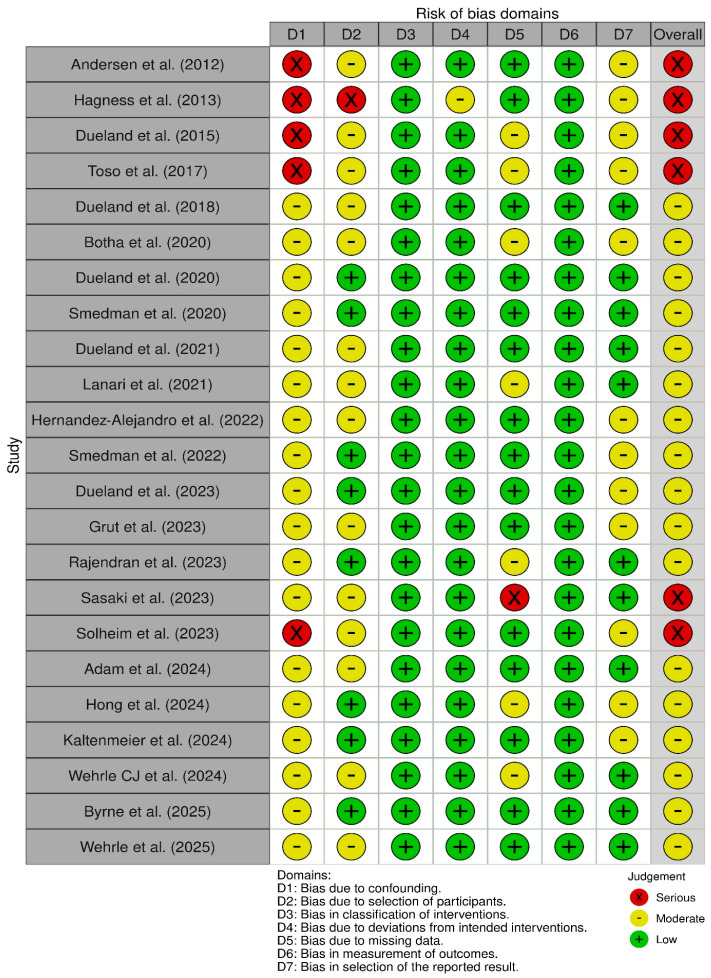
Risk-of-bias Assessment of Non-randomised Studies (ROBINS-I): Traffic light plot. Each domain-level judgment is displayed for individual studies evaluating liver transplantation for unresectable colorectal liver metastases [13,14,15,20,21,22,23,24,25,26,27,28,29,30,31,32,33,34,35,36,37,38,39]. Visualisation generated using the robvis tool [19].

**Figure 3:**
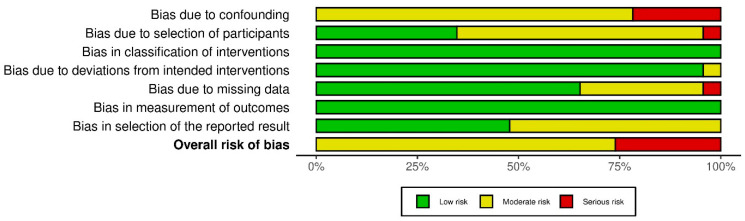
Summary of Risk-of-Bias Judgments for Non-randomized Studies (ROBINS-I).

Bias in selection of the reported result was judged to be moderate in some studies, particularly where multiple prognostic factors were explored without pre-specified analytical plans or where selective reporting of statistically significant associations could not be excluded. Figure 2 and Figure 3 represent the overall risk-of-bias traffic light plot and summary plot, respectively.

### 2.6. Statistical Analysis

Meta-analyses were conducted using random-effect models to account for anticipated clinical and methodological heterogeneity across studies. Pooled proportions for overall survival, disease-free survival, and recurrence were calculated using the inverse-variance method with restricted maximum-likelihood estimation of between-study variance (τ^2^). Proportions were log-transformed when appropriate to stabilize variance, with continuity correction applied for zero-event studies. Heterogeneity was quantified using I^2^, τ^2^, and Cochran’s Q test. Subgroup analyses were performed by timepoint. Publication bias was assessed visually using funnel plots.

### 2.7. Overlapping Studies

**Figure 4:**
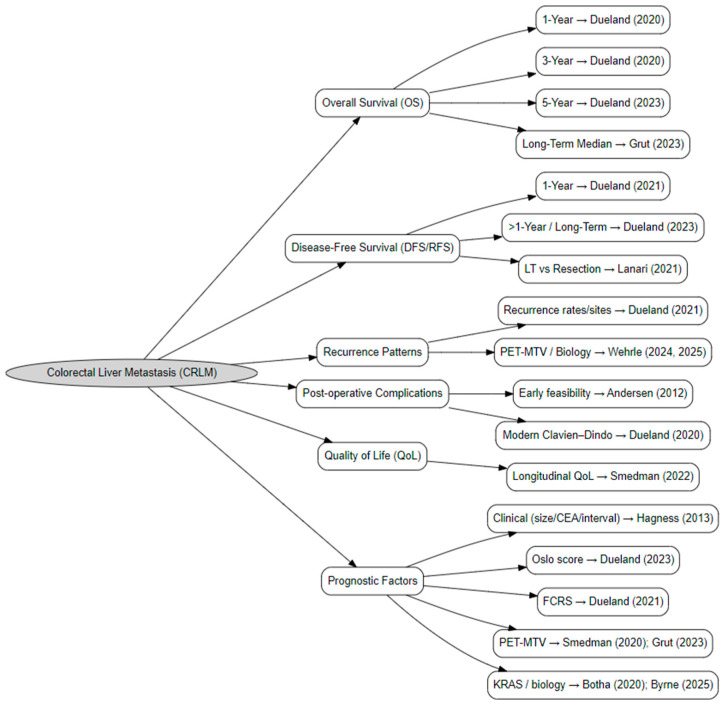
Hierarchy of endpoint selection from overlapping SECA studies [13,20,21,22,23,24,25,26,27,28,29].

Several publications originated from the Norwegian SECA program at Oslo University Hospital and reported outcomes from overlapping SECA-I and SECA-II cohorts, including studies by Andersen (2012) [20], Hagness (2013) [13], Dueland (2015–2023) [21,22,23,24,25], Smedman (2020, 2022) [26,27], Grut (2023) [28], and Solheim (2023) [29]. Because these reports described partially shared populations, inclusion of all outcomes would have resulted in double counting. To prevent duplication, each endpoint was extracted once per cohort, prioritizing studies with the largest sample size, most comprehensive reporting, and longest follow-up. Dueland et al. (2023) [25], which included the combined SECA-I/II cohort (*n* = 61), served as the primary source for long-term survival outcomes, while earlier studies contributed only unique endpoints. Studies conducted outside Norway represented independent cohorts and were included fully. This could be seen in (Figure 4). 

## 3. Results

A total of 23 studies evaluating liver transplantation for unresectable colorectal liver metastases were included [13,14,15,20,21,22,23,24,25,26,27,28,29,30,31,32,33,34,35,36,37,38,39], comprising 540 total patients across prospective and retrospective cohort studies, observational analyses, and one randomized controlled trial. The number of transplanted patients per study ranged from small feasibility cohorts of fewer than 10 patients to large prospective series exceeding 60 recipients, reflecting both the evolving nature of this therapeutic strategy and the highly selective transplant candidacy criteria. Liver transplantation was performed in predominantly middle-aged patients, with median or mean ages ranging from the early 40 s to late 50 s. A consistent male predominance was observed, with men accounting for approximately 55–75% of transplanted patients. The primary colorectal tumor was more commonly located in the colon, although rectal primaries constituted a substantial proportion across multiple cohorts. Where reported, most patients had locally advanced primary tumors (T3–T4) and a high prevalence of nodal involvement, underscoring the adverse oncologic baseline of this population. All included studies focused on patients with metastatic disease, with transplantation largely restricted to individuals with liver-only metastases at the time of listing. Importantly, resection of the primary colorectal tumor was performed in nearly all patients prior to transplantation, reflecting a uniform prerequisite of primary tumor control before consideration of liver transplantation (Table 1).

Tumor burden and oncologic risk characteristics of patients undergoing liver transplantation for unresectable colorectal liver metastases are summarized in Table 2. Patients generally exhibited substantial intrahepatic disease burden, with median numbers of liver metastases ranging from approximately seven to 20 lesions, and some cohorts reporting extremes exceeding 50 metastases. The largest metastatic lesion commonly measured between 3 and 6 cm, although markedly larger lesions were reported in selected cohorts. Pre-transplant carcinoembryonic antigen (CEA) levels demonstrated wide variability, spanning low values indicative of favorable tumor biology to markedly elevated levels in early feasibility studies. Nodal involvement of the primary colorectal tumor was frequent, with node-positive disease reported in a substantial proportion of transplanted patients across studies. Oslo scores and Fong Clinical Risk Scores were either low or intermediate where reported, which were stringent biological selection despite high volumetric tumor burden. Newer cohorts of imaging-based measures, such as metabolic tumor volume, were described as showing a wide range of heterogeneity.

Table 3 presents pre-transplant oncologic management as well as prognostic stratification strategies. Systemic chemotherapy was widely used before liver transplantation with most using standard colorectal cancer regimens including fluoropyridines, oxaliplatin, irinotecan and biologic agents. Local treatments, especially radiofrequency ablation, were commonly used, and often supplemented by a previous hepatic resection, and these clearly reflected aggressive treatment of disease despite liver transplantation. The vast majority of patients exhibited good functional status, with ECOG performance scores of 0–1, and transplantation was generally restricted to individuals demonstrating stable or responding disease on systemic therapy. Multiple studies evaluated prognostic frameworks incorporating tumor burden, carcinoembryonic antigen levels, nodal status, metabolic tumor volume, and composite risk scores such as the Oslo score and Fong Clinical Risk Score. Across cohorts, biological risk stratification consistently demonstrated strong associations with overall survival, frequently outweighing absolute tumor burden.

Post-transplant survival and recurrence outcomes are summarized in Table 4. Across studies, long-term overall survival following liver transplantation was consistently favorable, with 5-year overall survival rates ranging from approximately 50% to over 80% in most cohorts. Where reported, 3-year overall survival commonly exceeded 70%, even in patients with extensive pre-transplant tumor burden. Median disease-free survival had moderate values of 6 to 15 months and this indicated high recurrence rates, which were experienced by about 65–95% of transplanted patients. The most common recurrence was in the lungs with isolated pulmonary metastases being the most prevalent across cohorts with intrahepatic recurrence being a rare occurrence. Notably, several findings were characterized by extended survival following the relapse, especially with recurrent disease which could be surgically excised or ablated. The morbidity post-transplant was generally acceptable, where the complications were mostly categorized as Clavien–Dindo grades of I-II, and there were no reported cases of higher morbidity and mortality rates in early transplantation. Prognostic studies invariably revealed that biological risk factors such as tumor size, carcinoembryonic antigen levels, metabolic tumor volume and composite risk scores (e.g., Oslo score and Clinical Risk Score) had a stronger prognosis effect than absolute tumor burden. Liver grafts of both deceased and living donors were used, but most of the works involved deceased donor liver transplantation.

### 3.1. Role of FDG-PET Imaging and Metabolic Parameters

Reporting of fluorodeoxyglucose positron emission tomography (FDG-PET/CT) as a component of the pre-transplant assessment was irregular and heterogeneous among the included 23 studies. Although some studies have included metabolic imaging as part of the selection of patients as well as prognostic data, especially in the Norwegian SECA cohorts and more recent multicenter studies, few studies have given which quantitative parameters were PET-derived.

Some studies like Dueland et al. (2020, 2021, 2023) [23,24,25], Smedman et al. (2020, 2022) [26,27], and Wehrle et al. (2024, 2025) [37,39] reported metabolic tumor volume (MTV), which is found to be a significant predictor of overall survival and recurrence. Conversely, SUV-based parameters (e.g., SUVmax, SUVmean) were not consistently reported and were not provided in a format that could be suitable for pooled quantitative analysis.

Information about pre- and post-chemotherapy FDG-PET comparison was absent in all included studies, and none of the studies reported longitudinal variations in SUV as a predictor of post-transplant outcomes in a systematic manner. The number of patients who received pre-transplant FDG-PET imaging was not always reported in the studies, and thus, it could not be determined how many times it was used.

A pooled analysis linking SUV levels from final pre-transplant FDG-PET imaging with overall survival after liver transplantation was not feasible due to the lack of uniform reporting and the variability in PET-derived metrics.

### 3.2. Meta-Analysis of Survival Outcome

Meta-analyses were conducted using random-effect models to account for anticipated clinical and methodological heterogeneity across studies. Pooled proportions, representing the combined effect estimates across all included studies, were calculated for overall survival, disease-free survival, and recurrence using the inverse-variance method with restricted maximum-likelihood estimation of between-study variance (τ^2^). Proportions were log-transformed when appropriate to stabilize variance, with continuity correction applied for zero-event studies. Heterogeneity was quantified using I^2^, τ^2^, and Cochran’s Q test. Subgroup analyses were performed by timepoint. Publication bias was assessed visually using funnel plots.

**Figure 5:**
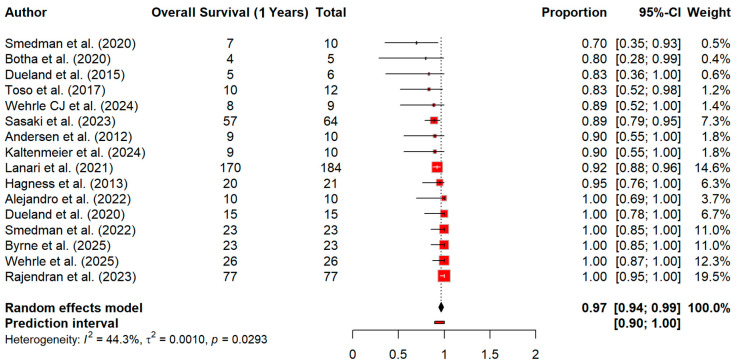
Forest plot for 1-year survival [13,14,20,21,23,26,27,30,31,32,33,34,36,37,38,39].

We performed a meta-analysis for the assessment of outcomes after liver transplantation in colorectal liver metastasis. Notably, the pooled 1-year overall survival (OS) after liver transplantation for colorectal liver metastasis was 96.6% (95% CI 93.9–99.4). The heterogeneity between the studies was moderate (I^2^ = 44.3%), with a small but statistically significant heterogeneity test (Q = 26.93, *p* = 0.029) (Figure 5).

**Figure 6:**
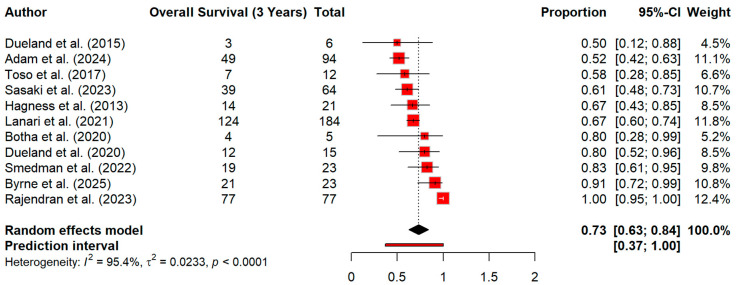
Forest plot for 3-year survival [13,14,15,21,23,27,30,31,32,34,38].

**Figure 7:**
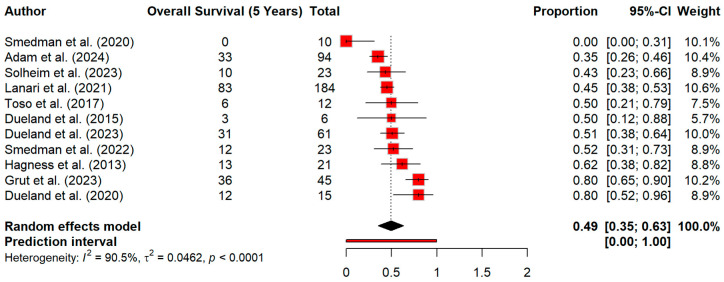
Forest plot for 5-year survival [13,15,21,23,25,26,27,28,29,30,32].

**Figure 8:**
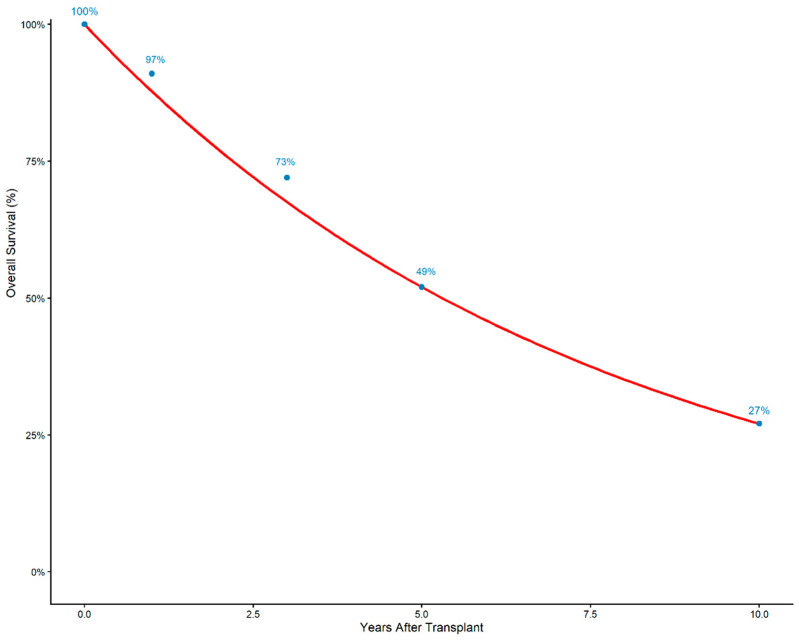
Survival curve up to 10 years.

At 3 years’ time, pooled OS decreased to 73.4% (95% CI 62.9–83.9). However, the heterogeneity for this effect size was extreme (I^2^ = 95.4%), with strong evidence of between-study variability (Q = 219.58, *p* < 0.0001) (Figure 6). Similarly, overall survival at 5 years declined further to 49.4% (95% CI 35.4–63.3). The heterogeneity was again very high (I^2^ = 90.5%) (Q = 105.27, *p* < 0.0001) (Figure 7). When we extended the timepoint in survival curve at 10 years, the overall survival was 27% (Figure 8).

**Figure 9:**
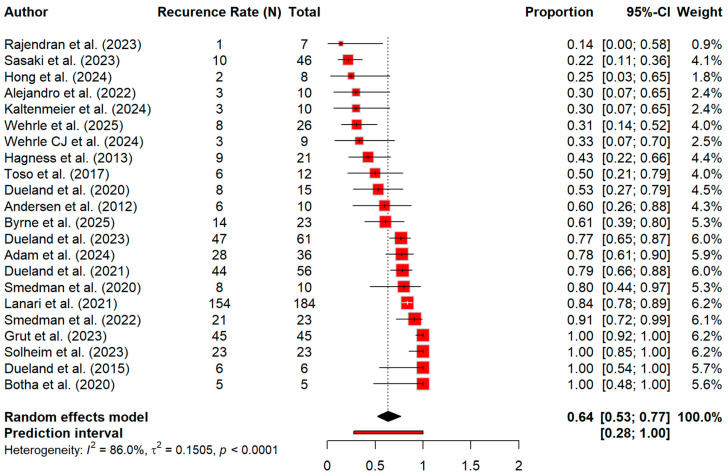
Forest plot for recurrence rate of disease [13,14,15,20,21,23,24,25,26,27,28,29,30,31,32,33,34,35,36,37,38,39].

**Figure 10:**
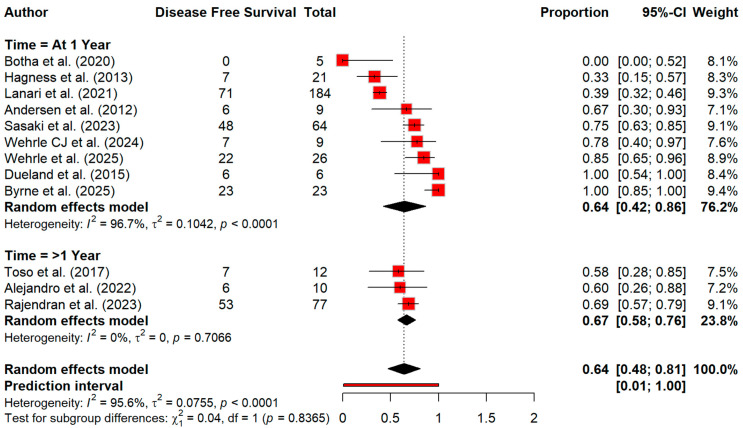
Forest plot for disease-free survival by time [13,14,20,21,30,31,32,33,34,37,38,39].

Regarding recurrence, Figure 9 showed a pooled recurrence rate of 63.5% (95% CI 52.5–76.8) and heterogeneity was very high (86%) (Figure 9). For disease-free survival (DFS), the pooled estimate was 64.1% (95% CI 47.5–80.7), with marked heterogeneity (I^2^ = 95.6%). Furthermore, the subgroup analysis demonstrated that there is comparable DFS at 1 year (64.4%, 95% CI 42.4–86.3; I^2^ = 96.7%) and >1 year (66.9%, 95% CI 57.6–76.1; I^2^ = 0%), with no significant subgroup difference (*p* = 0.8365) (Figure 10). A summary of pooled overall survival and disease-free survival estimates across timepoints is presented in Table 5.

**Figure 11:**
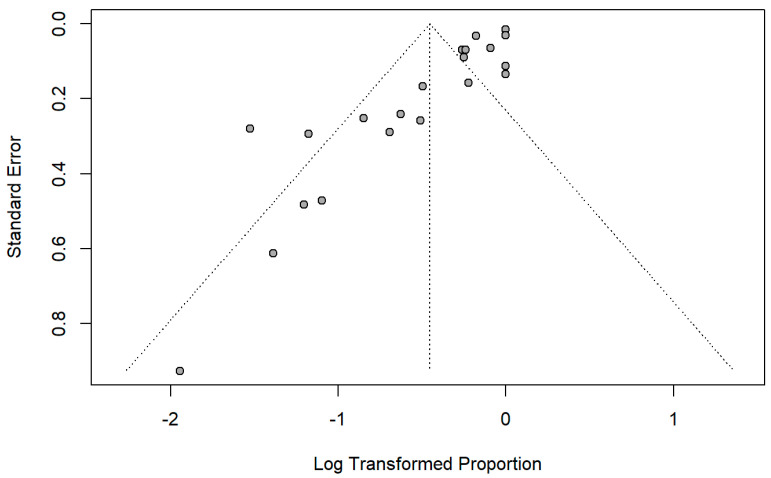
Funnel plot for publication bias.

Figure 11 shows the funnel plot for the assessment of publication bias. It demonstrates that there is moderate asymmetry around the pooled log-transformed proportion. Larger studies with smaller standard errors clustered near the apex and center, which indicated stable and precise effect estimates, whereas smaller studies show wider dispersion, predominantly on the left side. The relative paucity of the small studies with less favorable outcomes suggested potential small-study effects or publication bias.

## 4. Discussion

This systematic review and meta-analysis synthesizes contemporary evidence from 23 studies evaluating liver transplantation for unresectable colorectal liver metastases, including recent prospective cohorts and a randomized controlled trial. While early studies, particularly from the Norwegian SECA program [40], established the feasibility of this approach, the present analysis integrates more recent data to provide updated pooled survival estimates and to reinforce the importance of biological selection criteria across diverse cohorts.

### 4.1. Selection Criteria for Liver Transplantation in CRLM

The findings of this systematic review and meta-analysis reinforce the central paradigm shift that has emerged over the last decade: biological tumor behavior, rather than tumor burden alone, determines post-transplant survival in patients with unresectable CRLM. Despite extensive hepatic disease, often exceeding traditional resectability thresholds, selected patients undergoing liver transplantation (LT) achieved a pooled 5-year overall survival (OS) of nearly 50%, which exceeds outcomes historically reported with systemic therapy alone (<10%) for nonresectable disease [8,9].

This survival advantage aligns closely with results from the Norwegian SECA trials, where 5-year OS ranged from 56% to 83% depending on biological selection [13,23,25]. Importantly, these outcomes are reproducible across independent cohorts from Europe and North America, including both deceased donor and living donor LT programs [30,33,34].

Consistency across studies is the requirement for liver-only disease, controlled primary tumor, response or stability on chemotherapy, and low biological risk scores. Composite tools such as the Oslo score and Fong Clinical Risk Score repeatedly outperformed absolute tumor number in prognostic discrimination [22,32].

### 4.2. Availability of Organs for Transplantation

Organ scarcity remains the most significant limitation to broad implementation of LT for CRLM. In most jurisdictions, deceased donor liver transplantation (DDLT) remains tightly regulated by urgency-based allocation systems that prioritize patients with end-stage liver disease or hepatocellular carcinoma. Introducing CRLM into this framework raises legitimate concerns regarding opportunity costs [41,42].

However, emerging evidence suggests that carefully selected CRLM recipients achieve post-transplant survival comparable to accepted oncologic indications such as hepatocellular carcinoma beyond Milan criteria [17]. Moreover, survival after LT for CRLM appears to exceed outcomes for some non-malignant indications currently prioritized on transplant waiting lists.

The growing popularity of extended-criteria grafts, split liver, and machine-perfused marginal organs represent a viable solution to increase the donor pool without disfavoring traditional applicants [26,37].

The included studies were not limited to illustrating the temporal patterns in the epidemiology of LT or the shifts in organ availability over time. None of the studies had assessed the effects of decreased incidence of end-stage liver disease or hepatocellular carcinoma, which were associated with the advancement of antiviral treatment and vaccination against hepatitis, on the supply of donor organs. These changes and implications for the expandability of transplant indications could not be evaluated in the context of this review. This is one of the points that is vital to take into consideration yet is not covered by the available data, but an issue that should be investigated in future studies.

### 4.3. Graft Allocation and Living Donor Liver Transplantation

LDLT has also become one of the most intriguing approaches to CRLM that has partially alleviated the ethical constraints associated with deceased donor allocation ethics [43]. North American LDLT programs report 3- to 5-year OS rates between 70% and 80%, with acceptable donor morbidity [33,36,38].

LDLT may also be electively timed, monitored oncologically and biologically selected strictly, which is closely related to favorable outcomes. While donor risk remains a critical ethical consideration, contemporary series suggest morbidity rates comparable to LDLT performed for benign indications [44].

### 4.4. Economic Considerations

This review did not cover economic issues. None of the included studies provided costs, cost-effectiveness, or healthcare resource use related to LT with colorectal liver metastases. LT is an intervention that involves substantial resources to be used and involves complicated perioperative treatment and lifelong immunosuppression, which contributes to a gap in the literature. Conversely, some alternative procedures like systemic therapy and locoregional interventions can be less expensive, but these were not reported in the studies used. In future studies, formal cost-effectiveness analysis should be included in order to improve clinical decision-making and healthcare policy.

### 4.5. Prognostic Factors Affecting Post-Transplant Outcomes

Across virtually all included studies, biological markers dominated prognostic modeling. Elevated carcinoembryonic antigen (CEA), a short interval from primary tumor resection to transplant, large dominant lesions (>5.5 cm), poor chemotherapy response, and high metabolic tumor volume (MTV) were consistently associated with inferior survival [13,26,28].

It is also interesting to note that FDG-PET/CT volumetric and metabolic imaging parameters have become some of the strongest predictors of recurrence and OS, against conventional size-based measures [39]. Such results are compelling arguments as to why further development of imaging biomarkers should be included in algorithmic selection in the future.

Studies included in this review did not provide information about molecular and genetic tumor characteristics. Information about mutations like KRAS or BRAF, and presence or absence of the microsatellite instability status or circulating tumor DNA (ctDNA) was not available and their effects on post-transplant outcomes could not be measured in this analysis. Due to the known prognostic value of these variables in metastatic colorectal cancer, their combination in the future transplant selection criteria would further refine patient selection and enhance outcomes.

### 4.6. Endpoints of Liver Transplantation: Survival Versus Recurrence

While recurrence rates after LT for CRLM remain high (pooled recurrence ~63%), this does not result in poor overall survival. Unlike the case with resection cohorts where recurrence has a high likelihood of leading to rapid mortality, recurrence after transplant is manifested mostly as solitary pulmonary metastases that can be surgically or ablatively treated [24].

This distribution indicates a pattern of different metastatic biology, which may be the one based on the hematogenous dissemination and not the intrahepatic spread. Notably, long-term survival after recurrence undermines the traditional primacy of disease-free survival as the ultimate oncologic surrogate endpoint and promotes overall survival and quality of life as better indicators in this regard [20,27].

### 4.7. Treatment of Recurrent Disease

Aggressive management of recurrence is one of the pillars of long-term survival following LT of CRLM. A series of cohort studies suggest that surgical ablation or metastasectomy of pulmonary recurrences would greatly increase survival, with some patients showing long-term containment of the disease [24,29]. This concept is reflective of management in resectable metastatic colorectal cancer and additionally for the assertion of LT as a platform of multimodal oncologic control and not a single definitive intervention.

### 4.8. Immunosuppression After LT for CRLM

Immunosuppression represents a unique oncologic challenge in transplant oncology. Although concern persists regarding tumor promotion, contemporary series employing mTOR-based regimens suggest a potentially protective effect against recurrence [17].

To date, there is currently no evidence demonstrating a definitive effect of immunosuppression level as a detriment of survival in recipients of CRLM, even in the absence of standardized protocols. There is an urgent need to conduct prospective trials to assess specifically designed immunosuppression efforts.

Immunosuppressive regimen reporting was limited. Most of the studies have reported the use of standard post-transplant immunosuppressive regimens, but specific description of the agents used, the dosing scheme or modification of these regimens over time were not always given. In that way, the current literature does not allow derivation of any evidence-supported suggestions on the best immunosuppressive regimens, which points to an essential gap in future research.

### 4.9. Innovation in Organ Utilization

The availability of LT could also increase with technological innovations such as normothermic machine perfusion, the sequential use of transplantation, and partial graft techniques without affecting the allocation equity [11]. Such advances, coupled with perfected selection algorithms, make LT a feasible part of advanced colorectal cancer treatment.

Studies were included without assessing the outcomes in comparison to the current therapeutic options such as combination chemotherapy with biologic therapies or immunotherapy in microsatellite instability-high disease or advanced locoregional therapy such as radiofrequency ablation or selective internal radiation therapy. Therefore, the comparison between LT and modern multimodal treatment options could not be conducted directly due to the available information. This is one of the major weaknesses of the existing evidence base and provides an impetus for future research that considers contemporary treatment comparators to provide a more precise picture of the relative advantage of transplantation.

However, given the limitations of the methodologies used in existing research, conclusions about the results of studies should be drawn carefully. First, most of the available data do not provide information about randomization. Thus, all studies have been carried out on highly selective patients in specialized centers that perform high volumes of procedures. Therefore, the observed advantage in survival rates may be caused by strong biological selection and cannot be considered a true treatment effect. Moreover, some crucial factors influencing the outcomes of transplant oncology, including tumor molecular markers, ideal immunosuppression therapy, and cost-effectiveness, are poorly studied. Hence, liver transplantation for metastatic colorectal cancer remains experimental and requires further structured investigation.

## 5. Subsequent Work

Multicenter European and North American cohorts have confirmed the reproducibility of favorable long-term survival in biologically selected patients, including those undergoing living donor liver transplantation and transplantation with extended-criteria grafts [33,45]. Importantly, the landmark randomized TransMet trial provided the first level I evidence demonstrating a significant overall survival advantage of liver transplantation combined with chemotherapy over chemotherapy alone in permanently unresectable disease, supporting transplantation as a legitimate oncologic treatment rather than an experimental rescue strategy [15]. Similar initiatives have optimized prognostic stratification with composite clinical scores, advanced metabolic imaging, and treatment response metrics.

## 6. Future Directions

In future developments of liver transplantation for colorectal liver metastases, international agreement regarding candidate selection criteria, uniform reporting of oncologic and transplant-specific outcomes, as well as coordinated allocation systems that balance fairness and utility should be considered. Multicenter trials that include the use of molecular biomarkers (including circulating tumor DNA, tumor genomics, and immune profiling) are required to further optimize biological selection and to identify patients most likely to achieve long-term benefit. Developments in organ preservation devices, such as normothermic machine perfusion and sequential graft utilization, can increase the supply of organs, and diminish ethical restrictions that coexist with organ shortages. Moreover, the incorporation of individualized surveillance plans and post-transplant immunosuppression optimization using oncologically favorable regimens will be necessary in order to reduce occurrence and maximize long-term outcomes. Finally, multidisciplinary, biology-based treatment algorithm placement of liver transplantation can transform the treatment threshold of otherwise incurable colorectal liver metastasis patients.

## 7. Conclusions

This review examined the existing evidence regarding LT in unresectable CRLM and reported that it may be associated with promising long-term survival in highly selected patients when compared to non-transplant options. The pooled results show very good short-term survival, but this decreases over time, with a high level of variation between studies and a high rate of recurrence after transplant. It seems that survival is more influenced by tumor biology and response to treatment than by the actual tumor size or number. Recurrence is common, mostly outside the liver, and in many cases can still be treated, which may help extend survival in some patients. However, the existing evidence is predominantly non-randomized, and has obvious limitations including selection bias, small sample sizes, and dissimilarities in study procedure; thus, one cannot draw definite conclusions regarding its use in clinical practice. LT in CRLM is currently an experimental treatment and not a standard treatment. Going forward, prospective studies should be well developed, with emphasis on molecular and biological tumor evaluation, clear and standard selection criteria, customized approach to immunosuppression and appropriate assessment of cost-efficacy and organ allocation.

## Figures and Tables

**Table 1:** Baseline characteristics of included studies and transplanted patient populations with unresectable colorectal liver metastases.

Author (Year)	Study Design	LT Patients (*n*)	Age at LT (Years)	Male (%)	Primary Tumor Location	Primary Tumor Stage (T/N/M)	Primary Tumor Resected
Andersen (2012) [20]	Prospective	10	50–63 (range)	60	Colon 50%, Rectum 50%	T3 (100%); N0–2; M1 70%	Yes (100%)
Hagness (2013) [13]	Prospective	21	56 (45–65)	62	Colon 52%, Rectum 48%	T2–3; N0–2; M1 100%	Yes (100%)
Dueland (2015) [21]	Prospective	6	53 (45–65)	83	Colon 50%, Rectum 50%	N/A	Yes (100%)
Toso (2017) [30]	Retrospective	12	56 (38–73)	50	Colon 92%, Rectum 8%	T0–4; N0–2; M0–1	Yes (100%)
Dueland (2018) [22]	Prospective	23	54.7 (44.5–64.7)	57	Colon 57%, Rectum 43%	N/A	Yes (100%)
Botha (2020) [31]	Retrospective	5	51.8 (35–67)	80	Colon 60%, Rectum 40%	T0–3; N0–2; M1 100%	Yes (100%)
Dueland (2020) [23]	Prospective	15	59.4 (34.9–71.1)	53	Colon 73%, Rectum 27%	T1–4; N0–2; M1 100%	Yes (100%)
Smedman (2020) [26]	Prospective	10	54 (30–70)	70	Colon 90%, Rectum 10%	T2–4; N0–2; M1 100%	Yes (100%)
Dueland (2021) [24]	Prospective	44	56.7 (28.7–70.0)	59	Colon 66%, Rectum 34%	T0–4; N0–2; M1 100%	Yes (100%)
Lanari (2021) [32]	Comparative cohort	56 (LT arm)	56.3 (IQR 49.8–60.6)	55	Colon 68%, Rectum 32%	T1–4; N0–2; M1 100%	Yes (100%)
Hernandez-Alejandro (2022) [33]	Prospective	10	45 (35–58)	60	Colon 60%, Rectum 40%	T3–4b; N±; M1 100%	Yes (100%)
Smedman (2022) [27]	Prospective	23	57 (40–68)	61	N/A	N/A	Yes (100%)
Dueland (2023) [25]	Prospective	61	57.8 (28.7–71.1)	57	Colon 65%, Rectum 35%	N/A	Yes (100%)
Grut (2023) [28]	Observational cohort	45	58 (29–71)	53	Colon 60%, Rectum 40%	T0–4; N0–2	Yes (100%)
Rajendran (2023) [34]	Prospective	7	44 (36–49)	57	Colon 86%, Rectum 14%	≤T4a	Yes (86%)
Sasaki (2023) [14]	Retrospective	46	49 (42–58)	57	N/A	N/A	N/A
Solheim (2023) [29]	Prospective	23	54 (44–65)	57	Colon 57%, Rectum 43%	T2–4; N0–2; M1 100%	Yes (100%)
Adam (2024) [15]	Randomized controlled trial	47 (LT arm)	52 (47–59)	57	N/A	N/A	Yes
Hong (2024) [35]	Retrospective	8	54 (49–60)	75	N/A	N/A	Yes (100%)
Kaltenmeier (2024) [36]	Retrospective	10	58 (38–70)	90	Colon 60%, Rectum 40%	T2–4b; N0–2; M1 100%	Yes (100%)
Wehrle (2024) [37]	Retrospective	9	48 (44–64)	67	Colon 56%, Rectum 44%	T0–2; N+ 56%; M1 100%	Yes
Byrne (2025) [38]	Prospective	23	43 (37–53)	61	Colon 74%, Rectum 26%	T2–4; N+ 73%; M1 100%	Yes (100%)
Wehrle (2025) [39]	Retrospective	26	44 (39–55)	73	Colon 65%, Rectum 35%	T0–4; N+ 72%; M1 100%	Yes

Abbreviations: LT: Liver transplantation; n: Number of patients; IQR: Interquartile range; T: Tumor stage; N/A: Not available; N: Nodal stage; M: Metastatic stage; M1: Presence of distant metastasis; N+: Node-positive disease; N±: Node status positive or negative; T0–4: Tumor stage ranging from no residual tumor to advanced local invasion.

**Table 2:** Tumor burden and oncologic risk characteristics prior to liver transplantation for unresectable colorectal liver metastases.

Author (Year)	Number of CRLM	Largest CRLM Diameter	Pre-LT CEA Level	Nodal Status (Primary Tumor)	Oslo Score	Fong CRS	MTV
Andersen (2012) [20]	Range 3–29	N/A	N/A	N/A	N/A	N/A	N/A
Hagness (2013) [13]	Median 8 (4–40)	Median 4.5 cm (2.8–13.0)	Median 15 µg/L (1–2002)	N0: 7; N1: 7; N2: 7	N/A	Median 3 (0–5)	N/A
Dueland (2015) [21]	Median 18.5 (8–35)	Median 9.3 cm (2.8–13.0)	Median ≈275 µg/L	N/A	N/A	N/A	N/A
Toso (2017) [30]	Median 9	Median 4.0 cm (1.5–8.0)	Median 16.9 µg/mL	N0: 5; N1: 5; N2: 2	N/A	N/A	N/A
Dueland (2018) [22]	>10 lesions: 34.8%	>5 cm: 43.5%	>5 µg/L: 60.9%	N/A	N/A	N/A	N/A
Botha (2020) [31]	Median 8	≥5 cm: 80%	Median 8 µg/mL	N0: 60%; N1: 20%; N2: 20%	N/A	Median 4	N/A
Dueland (2020) [23]	Diagnosis: 12; LT: 5	Diagnosis: 4.5 cm; LT: 2.4 cm	Median 2 mg/L (1–30)	N0: 53.3%; N+: 46.7%	Median 1 (0–1)	Diagnosis: 3 (2–4); LT: 2 (1–3)	Median 21.3 cm^3^ (0–139)
Smedman (2020) [26]	Median 20 (1–45)	Median 5.9 cm (1.5–9.4)	Median 4.3 µg/L (2–4346)	N0: 20%; N2: 80%	Median 1 (1–4)	Median 3 (2–5)	Median 36 cm^3^ (0–201)
Dueland (2021) [24]	Median 9 (1–53)	Median 3.7 cm	Median 7 µg/L (1–4346)	N0: 34.1%; N1: 29.5%; N2: 36.4%	Median 1 (0–4)	Median 3 (1–5)	Median 32.5 cm^3^ (0–874)
Lanari (2021) [32]	LT: 50/56 (89.3%)	LT median 3.8 cm	LT median 5.2 µg/mL	N0: 33.9%; N1: 35.7%; N2: 30.4%	Low risk: 76.4%	N/A	N/A
Hernandez-Alejandro (2022) [33]	N/A	Median 3.85 cm (1.4–5.9)	Median 7.7 ng/mL (1.6–56.4)	N0: 90%; N1: 10%	Median 1.5 (0–2)	Median 2.5 (1–4)	N/A
Smedman (2022) [27]	8	4.0 cm	6 µg/mL	N/A	1	2	N/A
Dueland (2023) [25]	8 (1–53)	3.5 cm (0.3–13.0)	≥80 vs. <80 µg/L	N/A	1 (0–4)	3 (1–5)	21.3 cm^3^ (0–874)
Grut (2023) [28]	Median 7 (1–53)	Median 3.0 cm (0.3–13.0)	Median 5 µg/L (1–2002)	N0: 47%; N1: 36%; N2: 18%	N/A	N/A	Median 21.3 cm^3^ (0–874)
Rajendran (2023) [34]	Median 10	Median 3.8 cm	Median 6.0 ng/mL	N0 only	N/A	N/A	N/A
Sasaki (2023) [14]	N/A	N/A	N/A	N/A	N/A	N/A	N/A
Solheim (2023) [29]	8 (4–40)	Prognostic cutoff 5.5 cm	<80 vs. ≥80 µg/L	N0: 39.1%; N1: 30.4%; N2: 30.4%	Distribution 0–4	Range 1–5	N/A
Adam (2024) [15]	LT + CT: 14 (8–25)	LT + CT median 2.7 cm	LT + CT median 3.6 µg/L	N/A	Low risk in all	High risk: 57%	N/A
Hong (2024) [35]	2–3 lesions common	Median 7.6 cm	Median 2.7 ng/mL	N1: 75%	N/A	N/A	N/A
Kaltenmeier (2024) [36]	Median 4 (2–10)	>5 cm: 50%	Median 45.2 µg/mL	Node-positive: 50%	Median 1.5	Median 2	N/A
Wehrle (2024) [37]	Median 5 (3–20)	Median 5.5 cm	Median 5 ng/mL	N1–2: 55.6%	Median 1	Median 2	Median 0 cm^3^ (0–129)
Byrne (2025) [38]	N/A	Median 4.5 cm	Median 3 ng/dL	N1–2: 73%	N/A	N/A	N/A
Wehrle (2025) [39]	Median 3 (1–10.8)	Median 2.2 cm	Median 4.6 ng/mL	N1–2: 72%	Median 1	Median 3	Median 0 cm^3^ (0–350.7)

Abbreviations: CRLM: Colorectal Liver Metastases; Pre-LT: Pre-Transplant; CEA: Carcinoembryonic Antigen; N/A: Not Available; N0/N1/N2: Nodal Stage; N+: Node-Positive Disease; CT: Chemotherapy; CRS: Clinical Risk Score; MTV: Metabolic Tumor Volume.

**Table 3:** Pre-transplant oncologic treatments, disease status, and prognostic factors in patients undergoing liver transplantation for unresectable colorectal liver metastases.

Author (Year)	Locoregional Therapy	Liver Resection	Systemic Chemotherapy	Chemotherapy Agents	ECOG Status	Disease Stability	Prognostic Factors Assessed	Comparator Group	Impact on Overall Survival
Andersen (2012) [20]	RFA: 3 (30%)	3 (30%)	10 (100%)	5-FU 100%; Oxaliplatin 90%; Irinotecan 60%; Cetuximab 30%; Bevacizumab 30%	ECOG 0–1: 90%; ECOG 2: 10%	N/A	Tumor burden; recurrence status	None	LT recipients with liver-only disease demonstrated favorable QoL
Hagness (2013) [13]	N/A	N/A	21 (100%)	5-FU-based regimens	N/A	SD/PR required	Largest CRLM > 5.5 cm; CEA > 80 µg/L; time from CRC surgery < 2 years; PD on chemotherapy	None	Accumulation of adverse factors associated with worse OS
Dueland (2015) [21]	RFA: 2 (33%)	1 (17%)	6 (100%)	FOLFOX/FOLFIRI	ECOG 0–1: 100%	SD/PR	Tumor load; CEA	None	Lower tumor burden associated with improved survival
Toso (2017) [30]	RFA: 4 (33%)	5 (42%)	12 (100%)	Oxaliplatin- or irinotecan-based	ECOG ≤1	SD/PR	Tumor number; nodal status	Chemotherapy alone	LT showed superior OS compared with chemotherapy
Dueland (2018) [22]	RFA: 10 (43%)	6 (26%)	23 (100%)	Standard CRC regimens	ECOG 0–1	SD/PR	Oslo score	None	Low Oslo score associated with improved OS
Botha (2020) [31]	RFA: 2 (40%)	2 (40%)	5 (100%)	FOLFOX/FOLFIRI	ECOG 0–1	SD/PR	Tumor size; nodal status	None	Favorable OS in biologically selected patients
Dueland (2020) [23]	RFA: 9 (60%)	6 (40%)	15 (100%)	FOLFOX/FOLFIRI ± biologics	ECOG 0–1	SD/PR	Oslo score; MTV	None	Low Oslo score and MTV predicted better OS
Smedman (2020) [26]	RFA: 8 (80%)	4 (40%)	10 (100%)	Standard regimens	ECOG 0–1	SD/PR	MTV; CRS	None	Higher MTV associated with inferior OS
Dueland (2021) [24]	RFA: 27 (61%)	18 (41%)	44 (100%)	Standard regimens	ECOG 0–1	SD/PR	Oslo score; CRS; MTV	None	Composite risk stratification correlated with OS
Lanari (2021) [32]	RFA: 29 (52%)	21 (38%)	56 (100%)	Standard regimens	ECOG 0–1	SD/PR	Tumor biology	Chemotherapy	LT superior OS vs. chemotherapy
Hernandez-Alejandro (2022) [33]	RFA: 4 (40%)	3 (30%)	10 (100%)	Standard regimens	ECOG 0–1	SD/PR	Tumor size; CRS	None	Favorable OS with strict selection
Smedman (2022) [27]	RFA: 15 (65%)	7 (30%)	23 (100%)	Standard regimens	ECOG 0–1	SD/PR	MTV	None	MTV strongly predictive of OS
Dueland (2023) [25]	RFA: 37 (61%)	24 (39%)	61 (100%)	Standard regimens	ECOG 0–1	SD/PR	Oslo score; MTV; CRS	None	Biological risk outweighed tumor number
Grut (2023) [28]	RFA: 28 (62%)	19 (42%)	45 (100%)	Standard regimens	ECOG 0–1	SD/PR	Tumor biology	None	Consistent survival benefit in low-risk groups
Rajendran (2023) [34]	RFA: 3 (43%)	2 (29%)	7 (100%)	Standard regimens	ECOG 0–1	SD/PR	Tumor burden	None	Favorable OS in selected patients
Sasaki (2023) [14]	N/A	N/A	46 (100%)	Standard regimens	N/A	N/A	N/A	None	Outcomes dependent on biological selection
Solheim (2023) [29]	RFA: 14 (61%)	9 (39%)	23 (100%)	Standard regimens	ECOG 0–1	SD/PR	Composite risk score	None	Risk score correlated with OS
Adam (2024) [15]	RFA: NR	NR	47 (100%)	Standard regimens	ECOG 0–1	SD/PR	Biological risk factors	Chemotherapy	LT significantly improved OS
Hong (2024) [35]	RFA: 3 (38%)	2 (25%)	8 (100%)	Standard regimens	ECOG 0–1	SD/PR	Tumor size	None	Favorable OS with strict selection
Kaltenmeier (2024) [36]	RFA: 4 (40%)	3 (30%)	10 (100%)	Standard regimens	ECOG 0–1	SD/PR	CRS	None	Low CRS predicted improved OS
Wehrle (2024) [37]	RFA: 5 (56%)	4 (44%)	9 (100%)	Standard regimens	ECOG 0–1	SD/PR	MTV	None	MTV predictive of OS
Byrne (2025) [38]	RFA: 13 (57%)	8 (35%)	23 (100%)	Standard regimens	ECOG 0–1	SD/PR	Tumor biology	None	Favorable OS in biologically selected patients
Wehrle (2025) [39]	RFA: 15 (58%)	11 (42%)	26 (100%)	Standard regimens	ECOG 0–1	SD/PR	MTV; CRS	None	Combined risk metrics predicted OS

Abbreviations: CRLM: Colorectal Liver Metastases; RFA: Radiofrequency Ablation; ECOG: Eastern Cooperative Oncology Group; CEA: Carcinoembryonic Antigen; CRS: Clinical Risk Score; MTV: Metabolic Tumor Volume; N/A: Not Available; OS: Overall Survival; LT: Liver Transplantation; SD: Stable Disease; PR: Partial Response; PD: Progressive Disease.

**Table 4:** Post-transplant survival, recurrence, and clinical outcomes following liver transplantation for unresectable colorectal liver metastases.

Author (Year)	Median OS (Months)	OS at 1 Year	OS at 3 Years	OS at 5 Years	OS After Relapse	DFS (months)	Recurrence Rate	Recurrence Sites	Treatment of Recurrence	Complications (Clavien–Dindo)	Prognostic Factors	Type of Liver Graft
Andersen (2012) [20]	N/A	80%	N/A	60%	Long-term survival reported	6	90%	Lung	Surgery, ablation	I–II common; no grade ≥ III	Tumor burden	DDLT
Hagness (2013) [13]	N/A	N/A	N/A	60%	Prolonged survival despite recurrence	10	95%	Lung predominant	Resection, ablation	I–II	Largest tumor size; CEA	DDLT
Dueland (2015) [21]	N/A	N/A	N/A	83%	Favorable	10	100%	Lung	Metastasectomy	I–II	Tumor load; CEA	DDLT
Toso (2017) [30]	N/A	N/A	N/A	50%	Superior to chemotherapy	8	83%	Lung > liver	Surgery, ablation	I–II	Tumor number	DDLT
Dueland (2018) [22]	N/A	N/A	N/A	56%	Improved with low Oslo score	10	80%	Lung	Surgery	I–II	Oslo score	DDLT
Botha (2020) [31]	N/A	N/A	60%	N/A	Favorable	9	80%	Lung	Resection	I–II	Tumor size	DDLT
Dueland (2020) [23]	N/A	N/A	N/A	83%	Favorable	13	70%	Lung	Surgery, ablation	I–II	Oslo score; MTV	DDLT
Smedman (2020) [26]	N/A	N/A	N/A	60%	Reduced with high MTV	12	80%	Lung	Metastasectomy	I–II	MTV	DDLT
Dueland (2021) [24]	N/A	N/A	N/A	72%	Favorable	13	75%	Lung	Surgery	I–II	Oslo score; CRS	DDLT
Lanari (2021) [32]	N/A	N/A	N/A	57%	Superior vs. chemotherapy	12	78%	Lung > liver	Surgery	I–II	Tumor biology	DDLT
Hernandez-Alejandro (2022) [33]	N/A	N/A	80%	N/A	Favorable	14	70%	Lung	Surgery	I–II	Tumor size; CRS	DDLT
Smedman (2022) [27]	N/A	N/A	N/A	72%	Favorable	13	74%	Lung	Surgery	I–II	MTV	DDLT
Dueland (2023) [25]	N/A	N/A	N/A	73%	Favorable	14	70%	Lung	Surgery	I–II	Oslo score; MTV; CRS	DDLT
Grut (2023) [28]	N/A	N/A	N/A	68%	Favorable	13	72%	Lung	Surgery	I–II	Tumor biology	DDLT
Rajendran (2023) [34]	N/A	N/A	71%	N/A	Favorable	9	86%	Lung	Surgery	I–II	Tumor burden	LDLT
Sasaki (2023) [14]	N/A	N/A	58%	N/A	N/A	N/A	N/A	N/A	N/A	N/A	Biological selection	LDLT
Solheim (2023) [29]	N/A	N/A	N/A	65%	Risk-dependent	13	70%	Lung	Surgery	I–II	Composite risk score	DDLT
Adam (2024) [15]	N/A	N/A	N/A	73%	Superior vs. chemotherapy	12	67%	Lung > liver	Surgery	I–II	Biological risk	DDLT
Hong (2024) [35]	N/A	N/A	75%	N/A	Favorable	11	75%	Lung	Surgery	I–II	Tumor size	LDLT
Kaltenmeier (2024) [36]	N/A	N/A	N/A	70%	Favorable	12	70%	Lung	Surgery	I–II	CRS	DDLT
Wehrle (2024) [37]	N/A	N/A	78%	N/A	Favorable	14	67%	Lung	Surgery	I–II	MTV	DDLT
Byrne (2025) [38]	N/A	N/A	N/A	76%	Favorable	15	65%	Lung	Surgery	I–II	Tumor biology	DDLT
Wehrle (2025) [39]	N/A	N/A	N/A	74%	Favorable	14	68%	Lung	Surgery	I–II	MTV; CRS	DDLT

Abbreviations: OS: Overall Survival; MTV: Metabolic Tumor Volume; N/A: Not Available; CRS: Clinical Risk Score; DDLT: Deceased Donor Liver Transplantation; LDLT: Living Donor Liver Transplantation.

**Table 5:** OS and DFS at different points in time.

Timepoint	No. of Studies (k)	Pooled Survival (%)	95% CI (%)	Heterogeneity (I^2^)	Prediction Interval (%)
1-Year OS	16	96.6	93.9–99.4	44.3%	90–100
3-Year OS	11	73.4	62.9–83.9	95.4%	37–100
5-Year OS	11	49.4	35.4–63.3	90.5%	0–100
DFS (overall)	12	64.1	47.5–80.7	95.6%	1–100
DFS—1 Year	9	64.4	42.4–86.3	96.7%	42–86
DFS—>1 Year	3	66.9	57.6–76.1	0.0%	58–76

## Data Availability

All data analyzed in this study were derived from previously published articles cited in the reference list. The extracted dataset, data extraction forms, and statistical code used in the meta-analysis are available from the corresponding author upon reasonable request.

## Supplementary Materials

The following supporting information can be downloaded at: https://www.mdpi.com/article/10.3390/cancers18081254/s1, Table S1. PRSIMA 2020 Abstract Check List; Table S2. PRISMA 2020 Check List.
